# Implementation of Universal Newborn Screening for Severe Combined Immunodeficiency in Singapore While Continuing Routine Bacille-Calmette-Guerin Vaccination Given at Birth

**DOI:** 10.3389/fimmu.2021.794221

**Published:** 2022-01-03

**Authors:** Su-Wan Bianca Chan, Youjia Zhong, Soon Chuan James Lim, Sherry Poh, Kai Liang Teh, Jian Yi Soh, Chia Yin Chong, Koh Cheng Thoon, Michaela Seng, Ee Shien Tan, Thaschawee Arkachaisri, Woei Kang Liew

**Affiliations:** ^1^ Rheumatology and Immunology Service, Department of Pediatric Subspecialties, Kadang Kerbau (KK) Women’s and Children’s Hospital, Singapore, Singapore; ^2^ Duke-National University of Singapore (NUS) Medical School, National University of Singapore, Singapore, Singapore; ^3^ Khoo Teck Puat – National University Children’s Medical Institute, National University Health System, Singapore, Singapore; ^4^ Department of Paediatrics, Yong Loo Lin School of Medicine, National University of Singapore, Singapore, Singapore; ^5^ Yong Loo Lin School of Medicine, National University of Singapore, Singapore, Singapore; ^6^ Biochemical Genetics and National Expanded Newborn Screening, Department of Pathology and Laboratory Medicine, Kadang Kerbau (KK) Women’s and Children’s Hospital, Singapore, Singapore; ^7^ Infectious Diseases Service, Department of Pediatric Subspecialties, Kadang Kerbau (KK) Women’s and Children’s Hospital, Singapore, Singapore; ^8^ Lee Kong Chian School of Medicine, Nanyang Technological University, Singapore, Singapore; ^9^ Hematology Oncology Service, Department of Pediatric Subspecialties, Kadang Kerbau (KK) Women’s and Children’s Hospital, Singapore, Singapore; ^10^ Genetics Service, Department of Pediatrics, Kadang Kerbau (KK) Women’s and Children’s Hospital, Singapore, Singapore; ^11^ Paediatric Allergy Immunology Rheumatology Centre, Mount Elizabeth Novena Specialist Centre, Singapore, Singapore

**Keywords:** severe combined immunodeficiency (SCID), newborn screening (NBS), TREC, Bacille-Calmette-Guerin vaccination, Enlite Assay, BCG, Singapore

## Abstract

**Introduction:**

Severe Combined Immunodeficiency (SCID) is generally fatal if untreated; it predisposes to severe infections, including disseminated Bacille-Calmette-Guerin (BCG) disease from BCG vaccination at birth. However, delaying BCG vaccination can be detrimental to the population in tuberculosis-endemic regions. Early diagnosis of SCID through newborn screening followed by pre-emptive treatment with anti-mycobacterial therapy may be an alternative strategy to delaying routine BCG vaccination. We report the results of the first year of newborn SCID screening in Singapore while continuing routine BCG vaccination at birth.

**Method:**

Newborn screening using a T-cell receptor excision circle (TREC) assay was performed in dried blood spots received between 10 October 2019 to 9 October 2020 using the Enlite Neonatal TREC kit. Patients with low TREC had lymphocyte subset analysis and full blood count performed to determine the severity of lymphopenia and likelihood of SCID to guide further management.

**Results:**

Of the 35888 newborns screened in 1 year, no SCID cases were detected, while 13 cases of non-SCID T-cell lymphopenia (TCL) were picked up. Using a threshold for normal TREC to be >18 copies/μL, the retest rate was 0.1% and referral rate to immunologist was 0.04%. Initial low TREC correlated with low absolute lymphocyte counts (ALC), and subsequent normal ALC corresponded with increases in TREC, thus patients with normal first CD3+ T cell counts were considered to have transient idiopathic TCL instead of false positive results. 7/13 (54%) had secondary TCL (from sepsis, Trisomy 21 with hydrops and stoma losses or chylothorax, extreme prematurity, or partial DiGeorge Syndrome) and 6/13 (46%) had idiopathic TCL. No cases of SCID were diagnosed clinically in Singapore during this period and for 10 months after, indicating that no cases were missed by the screening program. 8/9 (89%) of term infants with abnormal TREC results received BCG vaccination within the first 6 days of life when TREC and ALC were low. No patients developed BCG complications after a median follow-up of 17 months.

**Conclusion:**

Newborn screening for SCID can be implemented while continuing routine BCG vaccination at birth. Patients with transient TCL and no underlying primary immunodeficiency are able to tolerate BCG vaccination.

## Introduction

Severe Combined Immunodeficiency (SCID) is a severe T-cell primary immunodeficiency caused by a heterogenous group of genetic defects which impair T cell maturation and development (1), and can also affect B and Natural Killer (NK) cell numbers and function, depending on the causative gene. The invasive and opportunistic infections which occur generally render the condition fatal if untreated. Successful hematopoietic Stem Cell Transplant (HSCT) can be curative, but outcomes are poorer once infections occur (2). Early diagnosis *via* screening of family members of positive cases improves outcomes by increasing the chances of successful transplant and reducing morbidity and mortality (2, 3), however only 20% of SCID cases have a positive family history (4).

Newborn screening (NBS) for SCID, using a T-cell receptor excision circle (TREC) assay on dried blood spots from a Guthrie card, was developed to enable pre-symptomatic diagnosis and improve outcomes (5, 6). First implemented in Wisconsin, USA in 2008 (7, 8),it has since been established in many healthcare systems worldwide. The production of a commercial Enlite TREC screening kit by PerkinElmer has facilitated implementation (9) by circumventing the logistical barrier of *de novo* development and validation of an in-house TREC assay. This Enlite TREC kit has been consistently effective in achieving early diagnosis of SCID in newborn screening programs in California (10), Catalonia (11), France (12) and Israel (13). While adoption of a validated kit facilitates standardization and quality control, each healthcare system needs to determine its own cut-off values based on what they consider to be an acceptable recall rate (14).

The healthcare systems that have published results of their newborn screening for SCID thus far do not routinely give Bacille-Calmette-Guerin (BCG) vaccination to all newborns at birth (11–15). Taiwan postponed BCG vaccination from 24 hours of age to 1 to 4 weeks of age in 2010 prior to starting nationwide newborn screening for SCID in 2012, and subsequently delayed it further to 5-8 months of age in 2016 to reduce potential BCG-related complications for those who are diagnosed with SCID (15).

Tuberculosis (TB) is endemic in Asia; of the 20 highest TB-burden countries in the world, 10 are in Asia (16). The World Health Organization recommends that countries with a high burden of TB or are neighboring highly TB endemic countries give BCG at birth to prevent TB meningitis and military TB in young children (16, 17). Singapore has a moderately high incidence of TB at 39 per 100000 people in 2018 (18), thus our national childhood immunization guidelines recommend routine administration of BCG vaccine to newborns prior to their first discharge from hospital. For term well babies, this is typically done at day 2 to 3 of life, on the same day that all blood for the Guthrie card is taken for Newborn Screening. For premature or unwell newborns, BCG vaccination is delayed until they are at least 35 weeks post-menstrual age, 2 kg body weight and ready for discharge from hospital. While this results in a BCG vaccination rate of almost 99% (18) and thus offers protection to the majority of the population from TB-related disease, disseminated BCG disease has occurred in a minority of patients with SCID who were inoculated with live BCG at birth, before SCID was diagnosed (19). This caused significant morbidity and mortality, and affected patients required adequate treatment before they could undergo curative HSCT. In contrast, asymptomatic BCG-vaccinated SCID patients started on anti-mycobacterial therapy early have been reported to have significantly less BCG complications before HSCT, less immune reconstitution syndrome post-HSCT, and less BCG-related mortality (20). Apart from SCID, other inborn errors of immunity (IEI) such as those causing Chronic Granulomatous Disease (CGD) and Mendelian Susceptibility to Mycobacterial Disease (MSMD) also predispose to severe disseminated BCG disease. However newborn screening tests are not currently available for any of these IEI apart from SCID.

Singapore started universal NBS for SCID in October 2019, as part of an expanded NBS program, without making changes to its existing national routine newborn BCG vaccination policy. Our strategy for preventing disseminated BCG disease in undiagnosed SCID patients was 1) early diagnosis of SCID through newborn screening and 2) early initiation of anti-mycobacterial therapy once diagnosis of SCID was made, following recommendations of the European Society of Immunodeficiency (21).

We report our experience of the first year of newborn screening for SCID in Singapore using the commercial Enlite Neonatal TREC Assay, and the clinical and epidemiological data which led to our decision to retain our practice of routine BCG administration at birth.

## Materials and Methods

### Population and Data Collection

SCID screening was added to an existing National Newborn Screening (NBS) Program for Inborn Errors of Metabolism that has been previously described (22). Briefly, dried blood spot (DBS) specimens were collected on Guthrie Cards from all infants born in Singapore whose parents had consented to testing. Blood from heel-pricks was collected from newborns after 24 hours of life, regardless of gestational age. In addition, we had a premature newborn protocol, adapted from the Clinical and Laboratory Standards Institute’s guidelines (22), that required the collection of 3 specimens — first sample at 24–72 hours of life, second at 2 weeks of life and third at 4 weeks of life. Extremely premature newborns were defined as those with a gestational age <32 weeks, all premature newborns as those <37 weeks, and term newborns as those ≥37 weeks. Regardless of hospital of birth, all DBS samples were processed at KK Women’s and Children’s Hospital Newborn Screening Laboratory. All DBS samples received between 10 October 2019 and 9 October 2020 were analyzed, with no exclusions. Anonymized demographic data (date of and age at sample collection, newborn gender, gestational age, birth weight) were collected based on information written on the Guthrie Card by the healthcare provider.

Newborns received routine BCG vaccination intradermally prior to first discharge from hospital. BCG strain and location of injection (left deltoid or left gluteal) differed depending on hospital of birth. BCG vaccination was typically given on the same day as DBS collection for well term babies, thus the TREC screening results were generally not available before BCG vaccination. Only babies in the Neonatal Intensive Care Unit or Special Care Nursery with prolonged stays had TREC results available before BCG vaccination. Newborns with known abnormal TREC results had their BCG vaccination delayed until lymphopenia had improved or resolved.

The data generated for this study was collected anonymously as part of a Clinical Audit on the outcomes of Newborn Screening for SCID in Singapore, in accordance with local ethical board regulations. Review by the Institutional Review Board in Singapore is not required as per local regulations. Specific informed consent was obtained from all individual participants included whose genetic evaluation was required as part of routine clinical practice. Data for **Supplementary Table 2** was collected as part of a previous retrospective study approved by the SingHealth Centralized Institutional Review Board (CIRB Reference Number: 2018/2205).

### Enlite Neonatal TREC Testing

The TREC assay was performed using the EnLite Neonatal TREC kit (Perkin Elmer, Turku Finland) according to the instructions provided. A single 1.5mm disc from each Guthrie filter card was directly punched into a 96-well plate using a 9-plate Panthera DBS puncher (2081-0010, PerkinElmer). DNA was eluted from each disk in the well with an Elution buffer and incubated, followed by PCR amplification and hybridization with a lanthanide target sequence specific probe. The detection of TREC, which when low is the biomarker for SCID, and beta-actin which is a ubiquitous housekeeping gene that acts as an internal control, was simultaneously measured by a PerkinElmer Victor Enlite fluorometer based on a time-resolved fluorescence energy transfer (TR-FRET) technology. Assay validation was performed as detailed in the Supplemental Methods.

### Selection and Verification of TREC Cut-Off Value

Prior to implementation of the TREC assay in the National Expanded NBS program, a prospective pilot study was conducted using 2,082 anonymized samples. The mean, median, 5^th^ and 95^th^ percentile of TREC values in this cohort were 113, 94, 33 and 246 copies/µL respectively.

**Figure 1:**
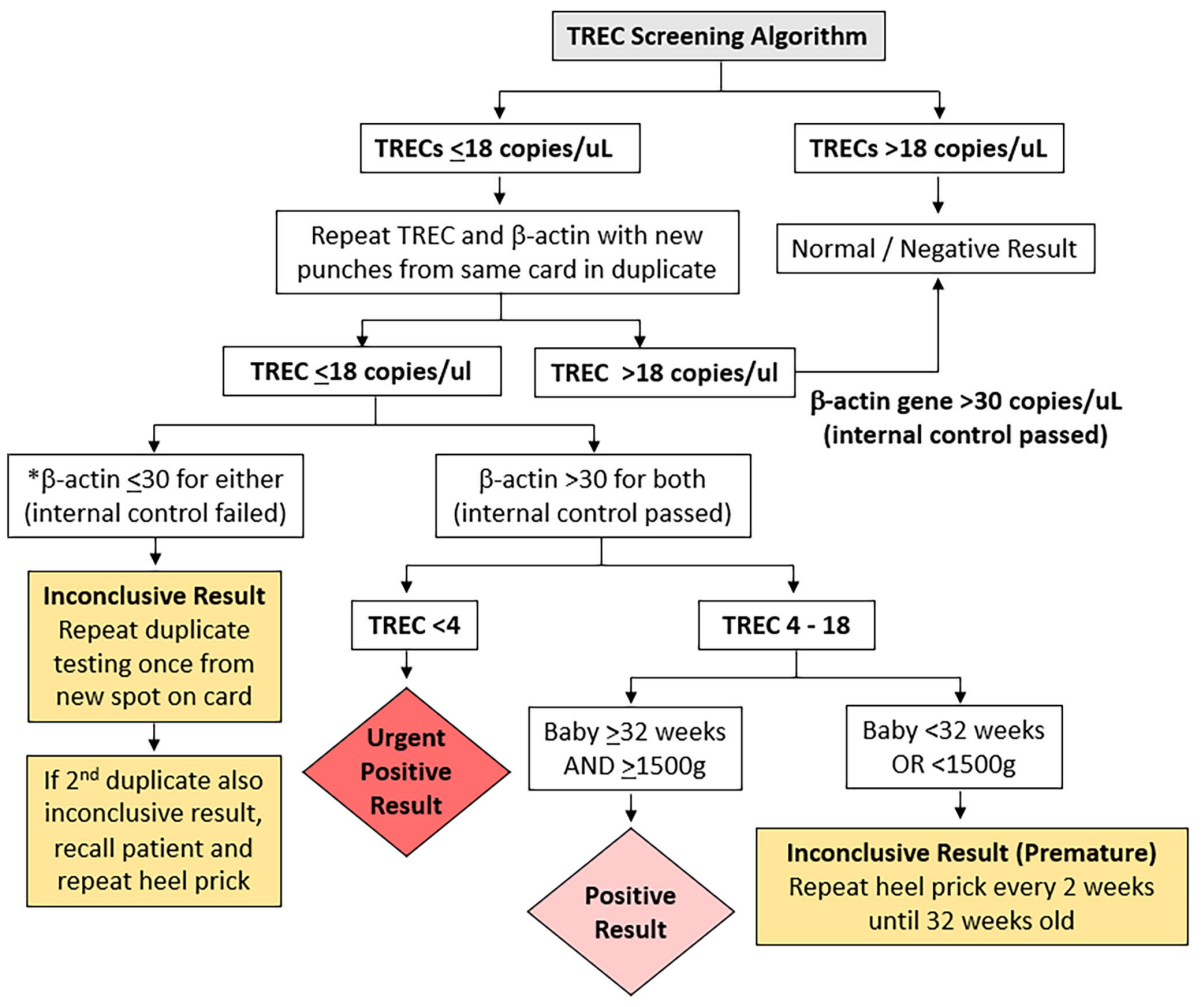
T cell receptor Excision Circle (TREC) screening algorithm. *if beta-actin gene ≤ 30 copies/uL the sample was considered of unsatisfactory quality.

In our selection of a TREC cut-off for our neonatal population we reviewed the CLIR (Collaborative Laboratory Integrated Reports) SCID database maintained at Mayo Clinic, Rochester, Minnesota, USA. CLIR is a consortium comprising over 270 NBS programs in 70 countries. Using this validated disease range values (113 cases in 2020, TREC values range: 0.21 (1^st^ percentile) – 24.88 copies (99^th^ percentile) for SCID and other types of Immunodeficiency syndromes) as a guide, we chose the target cut-off value of 18 copies/µL (1^st^ percentile of population study), taking into consideration the predicted effect on clinical utility, recall rates, false negative and false positive rates. In addition, we reviewed and compared several international SCID programs using the Enlite Neonatal TREC reagent kit (**Table 1**) (10–13, 15, 23, 24). To validate the chosen TREC cut-off value, a combination of anonymized USA CDC proficiency samples (2018 Q4, 10 samples), 20 samples from SCID patients and other dried blood spot samples from unaffected newborns were used. The range of TREC values in this validation study were as follows: positive cases (0-23 TREC copies/µL); normal samples: (24-107 copies/µL); beta-actin ranged from 3-6870 copies/µL. Three QC kit controls (0, low and high TREC) were added at the front and back end of each plate. All samples were correctly identified except for a sample from a patient with Combined Immunodeficiency from an underlying TTC7A mutation (TREC =23 copies/µL). We considered moving the TREC cut-off from 18 to 24 copies/µL, however the projected number of referrals based on our pilot study suggested that up to 2% of annual live births (40,000/year) could potentially be recalled for retesting and follow-up. Since there was a need to balance between specificity and sensitivity of the test, we decided to retain the initial TREC cut-off of 18 and review periodically at 6-month intervals. This TREC cut-off was implemented in conjunction with a screening algorithm adapted from the California Newborn Screening program (10) (**Figure 1**).

**Table 1:** Comparison of results from different centers that use the Enlite Neonatal TREC Kit.

Center	TREC Cutoff (copies/µL)	Number actually screened	TREC (+), repeat in duplicate (% of screened)	Abnormal TREC (% of screened)	Normal initial subsets [w% of TREC (+)]	True lymphopenia [% of TREC (+)]	Inconclusive [% of TREC (+)]	Population to screen (per year)	Yearly referral rate for abnormal TREC (% of screened)
**Dutch 2017, 2018** (23, 24)**^**	<40	1295	39 (3%)	21 (1.62%)	–	**-**	1 (3%)	–	1.62%
	<30	1272^	38 (3%) ^	7 (0.54%)	**-**	**-**	–	175181	0.54%
	<22	1295	2 (0.15%)	1 (0.08%)	–	–	–	–	0.08%
**Taiwan 2017** (15)	<36	290864	(4.24%)	–	**-**	**-**	**-**	–	–
	<26	290864	(1.94%)	–	**-**	**-**	**-**	–	–
	<25	213164	NR	10 (0.005%)	6 (60%)	4 (40%)	0 (0%)	200000	0.005%
	<23	290864	(0.95%)	–	**-**	**-**	–	–	–
**Israel 2017** (13)	<23	177277	561 (0.3%)	46 (0.02%)	11 (24%)	35 (76%)		185000	0.02%
**California 2019** (10)	≤18	3252156	NR	562 (0.02%)	339 (60%)	218 (39%)	5 (1%)	500331	0.02%
**France 2019** (12)	≤21	190517	430 (0.23%)	–	–	–	**-**	**-**	**-**
	≤10	190517	NR	165 (0.09%)	78 (56%)	62 (44%)	**-**	722960	0.09%
**Catalonia 2019** (11)	≤20	129,614	3108 (2.4%)	30 (0.02%)	9 (30%)	18 (60%)	3 (10%)	130903	0.02%
**Singapore 2021 (Current study)**	**≤18**	**35888**	**36 (0.1%)**	**13 (0.04%)**	**5 (38%)**	**7 (54%)**	**1 (8%)**	**39000**	**0.04%**

^Results taken from actual screening evaluation performed on 1272 cards in Blom M et al. (23). TREC, T cell excision circles; (+) positive; NR, not reported.

The bold values indicate that those data is from current study.

### TREC Screening Algorithm

The TREC screening algorithm is summarized in **Figure 1**. Samples whose TREC value was above the threshold of 18 copies/µL on a single DBS obtained at any age were considered normal. If the TREC was equal to or below the threshold of 18 copies/µL, a repeat punch for TRECs and a control beta-actin gene segment copy number determination was performed from the same specimen in duplicate. Any beta-actin reading below the threshold of 30 (indicating low PCR amplification) was considered inconclusive, and a third punch was obtained in duplicate on another DBS from the same card. If the third sample was also inconclusive, the patient was recalled for a repeat heel prick to obtain a fresh DBS. Samples whose TREC was ≤18 copies/µL and both beta-actin genes were >30 copies/µL were considered to be positive if the baby was ≥32 weeks gestational age and ≥1500g, and urgently positive if the TREC was <4 copies/µL regardless of the baby’s gestational age. If the TREC was between 4 to 18 copies/µL and the baby was <32 weeks or <1500g the result was deemed inconclusive (premature) and a repeat heel prick was performed every 2 weeks until the gestational age of 32 weeks.

Positive and urgent positive cases were immediately referred to the on-duty pediatric immunologist in KK Women’s and Children’s Hospital or National University Hospital for further clinical and immunological evaluation. Inconclusive premature cases were only referred if results were persistently positive. The retest rate after the first sample, patient recall rate for repeat heel prick, and referral rate to immunologist for positive results were calculated.

### Initial Clinical and Immunological Assessment

**Figure 2:**
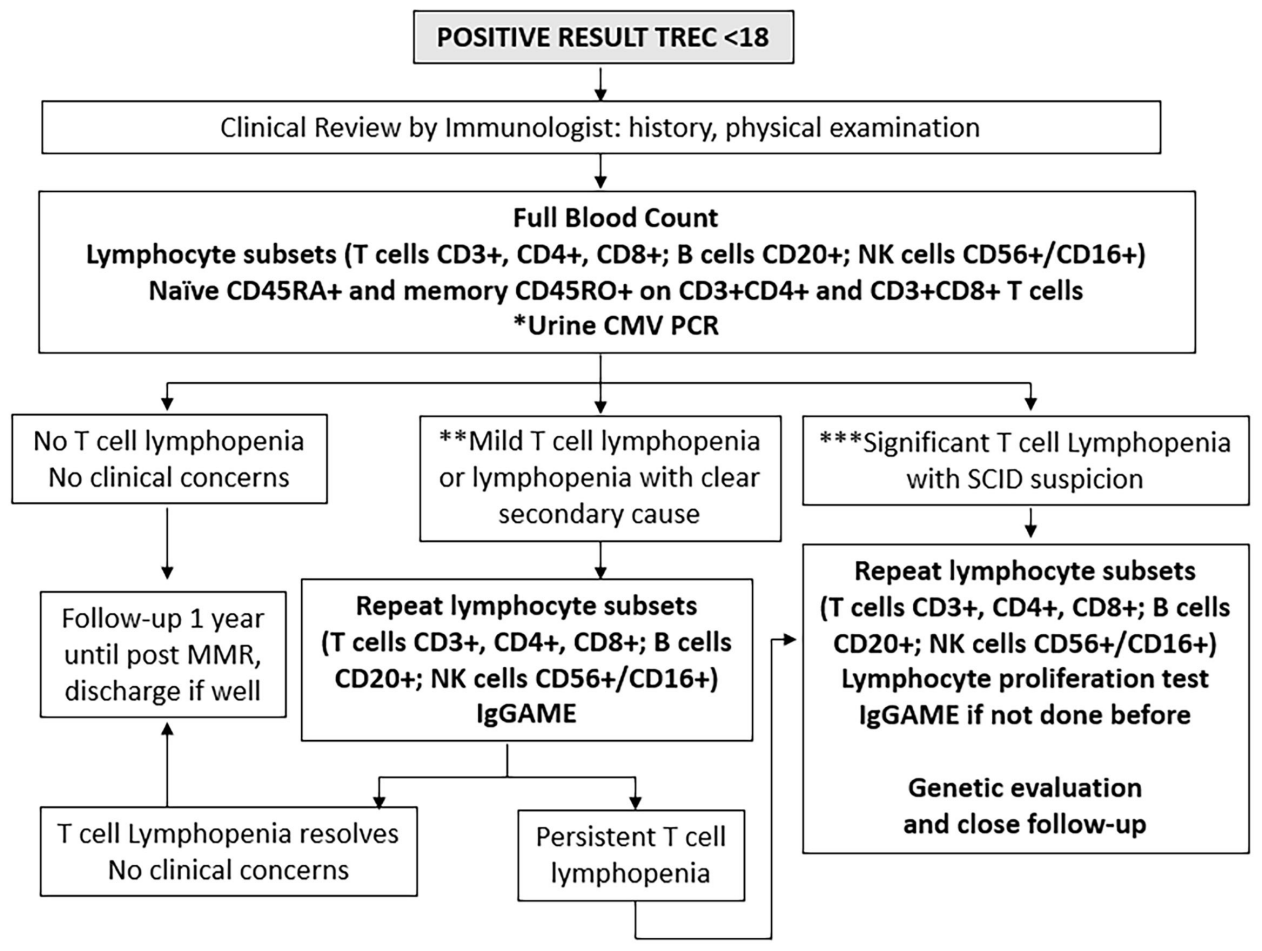
Immunological investigations for positive cases. *Urine Cytomegalovirus (CMV) Polymerase Chain Reaction (PCR) testing within 21 days to exclude congenital infection. **Mild T cell lymphopenia define as CD3+ below normal for age but > 300 cells/uL. ***Significant T cell lymphopenia CD3+ < 300 cells/uL. NK, natural killer cells; IgGAME, Immunoglobulin G, A, M, and E; MMR, mumps, measles, rubella vaccination.

All positive and urgent positive cases were recalled immediately for clinical review and immunological assessment (**Figure 2**). A thorough history and physical examination was performed as part of routine clinical evaluation, followed by liquid blood analysis of full blood count with differential (FBC) and lymphocyte subsets. For inconclusive cases, where either internal control failed or infants were extremely premature at <32 weeks gestational age or <1500g, a repeat DBS was obtained, and if persistently abnormal, then clinical evaluation, FBC and lymphocyte subsets were performed.

The lymphocyte subset determination of CD3+ T cells, CD3+CD4+ helper T cells, CD3+CD8+ cytotoxic T cells, together with whether CD3+CD4+ and CD3+CD8+ cells were of CD45RA+ naïve or CD45RO+ memory phenotypes, as well as CD20+ B cells and CD56+ or CD16+ (CD56+/CD16+) NK cells was run at a single laboratory at the National University of Singapore (7-parameter, 5-colour flow cytometry using fluorescent-tagged monoclonal antibodies and a FACS Canto II flow cytometer with FACSDIVA software from Becton Dickinson and Company, Franklin Lakes, NJ, and BD Biosciences, San Jose, CA), using age- and gender-adjusted normal ranges previously established in the local population (25).

Results were interpreted by pediatric immunologists at KK Women’s and Children’s Hospital, National University Hospital of Singapore, and Mt Elizabeth Novena Hospital, Singapore. Infants were considered to have significant TCL if they had <300 CD3+ T cells/µL or <20% CD3+CD4+CD45RA+ naïve helper T cells as defined by the Primary Immune Deficiency Treatment Consortium (26, 27), upon which full evaluation for possible SCID was immediately performed, subject to parental consent. Infants were considered to have mild lymphopenia if they had <1500 but >300 CD3+ T cells/µL; these were evaluated for leaky SCID and other causes of secondary lymphopenia. The cut-off of <1500 CD3+ T cells/µL for mild lymphopenia was chosen following the algorithm by Amatuni et al. in California, as TCL below this level would be more clinically significant and necessitate withholding of the rotavirus vaccine (10, 14). Infants with normal CD3+ T cells for age were followed up for at least 1 year. Urine cytomegalovirus (CMV) PCR was performed for most referred infants together with post-natal maternal CMV IgG status to exclude congenital CMV infection and provide guidance on breastfeeding. If initially abnormal, lymphocyte subsets were repeated until they normalized, at intervals according to clinician discretion. Immunoglobulin (Ig)G, IgA, IgM and IgE levels and lymphocyte proliferation tests (to Phytohemagglutinin (PHA), Concavalin A and anti-CD3 and anti-CD28 stimuli) were performed for cases with initial severe TCL and high suspicion for SCID or those with persistent lymphopenia at clinician discretion, in view of the cost and the large volume of blood required to be drawn for testing. Diagnoses were established by standard clinical testing. Genetic testing by next-generation sequencing (NGS) panel for primary immunodeficiency (PID) was performed as necessary using a commercial targeted gene panel from Fulgent or Invitae covering over 400 known PID-causing genes. Whole Genome Sequencing was done as a commercial test by Prevention Genetics if the NGS panel was not diagnostic. Genetic testing was only performed with informed consent after appropriate genetic counselling by a trained Genetic Counsellor. Cases without a clear secondary cause, birth defect, or syndrome were classified as idiopathic. TCL which resolved within a year was considered transient, while those that lasted more than a year were considered persistent (28). **Figure 2** outlines the immunological and genetic investigations performed in a step-wise manner for all positive cases.

## Results

### Demographics

**Figure 3:**
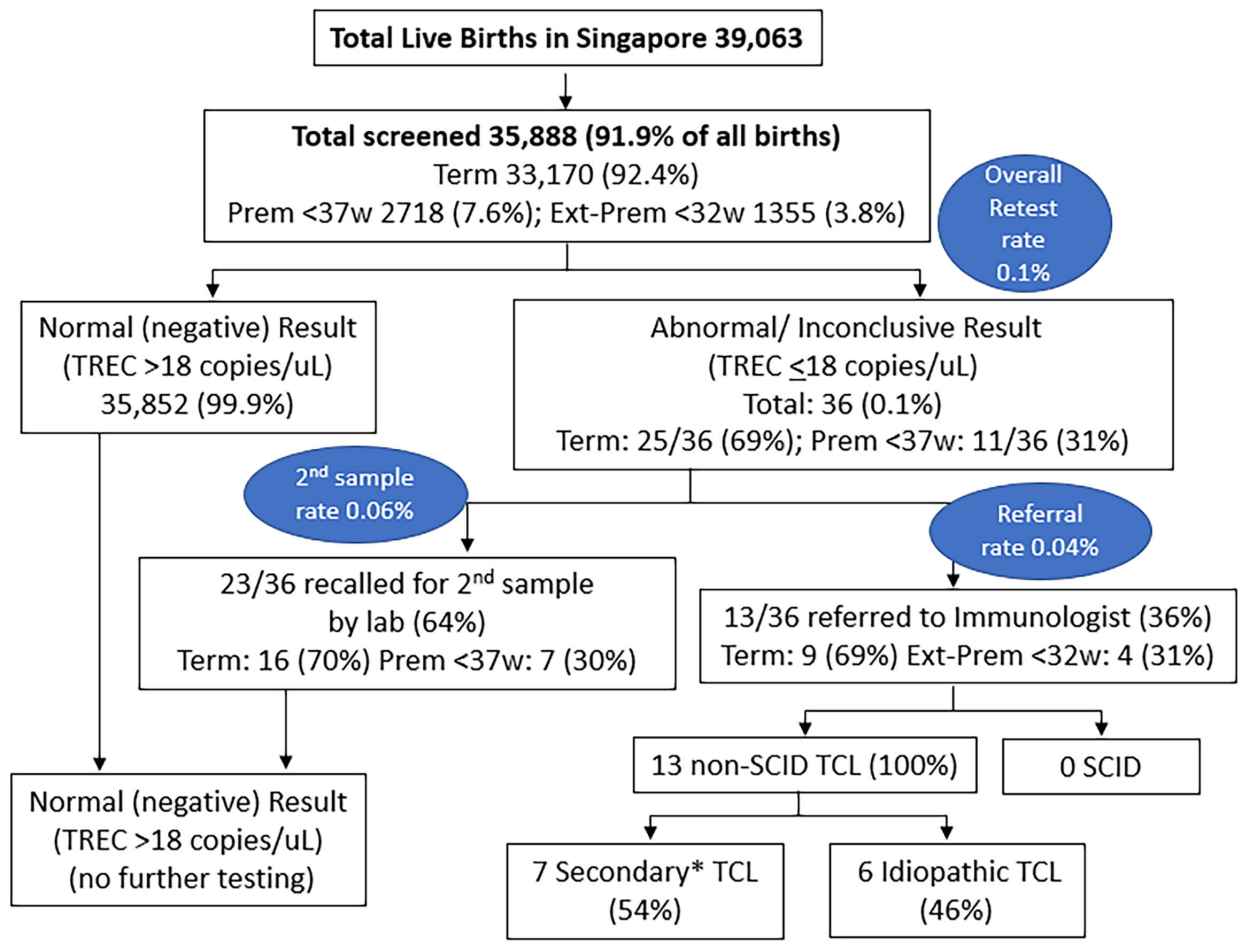
Results of SCID Newborn Screening in Singapore from 10 October 2019 to 9 October 2020. *7 Secondary TCL: 2 Sepsis, 1 Trisomy 21 with hydrops and stoma losses, 1 Trisomy 21 with chylothorax, 1 very premature with IUGR, 1 very premature, 1 partial DiGeorge Syndrome. Prem, premature; Ext-Prem, extremely premature; SCID, severe combined immunodeficiency; TCL, T-cell lymphopenia; TREC, T-cell receptor excision circle; w, weeks old.

Of the 39,063 births in Singapore between 10 October 2019 and 9 October 2020, 35,888 underwent SCID screening (a capture rate of 91.9%, **Figure 3**), while 3175 newborns did not participate in the voluntary program. Of those screened, 33,170 (92.4%) were term newborns with gestational age ≥ 37 weeks, while 2718 were premature < 37 weeks (7.6%), with 1355 (3.8%) being extremely premature < 32 weeks. A total of 36 (0.1%) had non-normal or persistently inconclusive TREC results, of which 69% were term and 31% were premature. 23 cases (0.06%) required a repeat heel prick with normal results on the repeat sample, while 13 cases (0.04%, 1 in 2760) were referred for immunological assessment and required lymphocyte subsets. Of the 13 newborns referred, 9 (69%) were term and 4 (31%) were extremely premature <32 weeks old – laboratory and clinical data are described in **Table 2**. There was no history of maternal immunosuppression or family history of primary immunodeficiency in any of the cases with abnormal TREC results.

**Table 2:** Results of 1 year of TREC Screening (10 October 2019 to 9 October 2020).

Case	GA*	Sex	Age+	TREC# (copies/µL)	ALC^ (x10^6^/L)	CD3+ (cells/µL) (%)	CD3+/CD4+ CD45RA+	Diagnosis	BCG vaccination	Resolution of TCL (age)/follow-up duration
1	26+2	M	3 d	10	2.33	–	–	Very Premature with pulmonary hypertension, severe BPD, post-natal CMV disease	Yes, at 1y 4m old, after TCL resolved	Yes (6 months)/22 months
			13 d	14	1.82	–	–			
			70 d	12	–	–	–			
			78 d	–	–	633 (36%)	87.4%			
			16 m	–	–	2157 (60%)	82.8%			
**2**	27+1	M	2 d	5	0.75	–	–	Very Premature with severe IUGR, Pulmonary hypertension with severe cystic BPD	No, died at 5 months old	Incomplete, died before resolution/5 months
			12 d	–	3.69	–	–			
			14 d	10	**-**	–	–			
			29 d	11	3.14	–	–			
			76 d	–	–	1773 (54%)	94.0%			
**3**	29+1	F	5 d	6	0.60	–	–	Very Premature ELBW Trisomy 21 with SGA, imperforate anus s/p colostomy, with chylothorax	No, died at 2 months old	Incomplete, died before resolution/2 months
			15 d	7	0.50	–	–			
			28 d	10	0.94	1265 (73%)	88.7%			
			43 d	11	1.29	893 (66%)	–			
			57 d	–	2.23	2096 (53%)	–			
**4**	32+2	M	6 d	7	0.47	–	–	Premature Trisomy 21 with SGA, fetal hydrops, ileal atresia s/p stoma, GI losses	Yes, at 42 days old, after TCL resolved	Yes (29 days)/22 months
			14 d	14	0.96	–	–			
			29 d	50	3.04	2149 (65%)	82.4%			
**5**	37+1	M	2 d	7	0.00	–	–	Sepsis-related secondary TCL (E Coli bacteremia, meningitis)	Yes, at 21 days old after TCL resolved	Yes (Likely by 7 days)/19 months
			7 d	–	4.86	–	–			
			14 d	89, 97	–	declined				
			1 y	–	5.07	2724 (66%)	–			
**6**	37+6	F	2 d	15	–	–	–	Persistent idiopathic TCL of unclear cause	Yes, at 2 days old	No/12 months
			11 d	31,25, 51	3.15	clotted	–			
			18 d	–	–	1043 (53%)	86.3%			
			6 m	11	2.64	881 (39%)	–			
			10 m	12	1.82	683 (44%)	–			
**7**	38+3	F	2 d	IC	–	–	–	Transient idiopathic TCL	Yes, at 1 day old	Yes (17 days)/13 months
			10 d	14	–	–	–			
			17d	–	5.02	2090 (47%)	80.0%			
**8**	38+4	M	3 d	6	–	–	–	Transient idiopathic TCL	Yes, at 3 days old	Yes (13 days)/20 months
			13 d	92, 75	7.11	5525 (73%)	73.6%			
**9**	39+0	F	1 d	12	–	–		Transient idiopathic TCL	Yes, at 1 day old	Yes (9 days)/22 months
			9 d	–	7.77	3957 (81%)	72.4%			
**10**	39+1	M	1 d	–	2.17	–	–	22q11 Syndrome with transient TCL	Yes, at 6 days old	Yes (19 days)/10 months
			3 d	IC	–	–	–			
			12 d	7	–	–	–			
			19 d	–	3.58	2248 (51%)	86.0%			
**11**	40+0	F	3 d	7	–	–	–	Sepsis-related secondary TCL (low platelets, culture negative)	Yes, at 3 days old	Yes (7 days)/14 months
			7 d	94	5.83	3093 (67%)	71.1%			
**12**	40+0	F	2 d	1	–	–	–	Initial severe TCL, Transient idiopathic TCL	Yes, 1 day old	Yes (6.5 months)/13 months
			8 d	–	1.52	124 (12%)	61.3%			
			14 d	–	–	210 (15%)	–			
			62 d	IC	–	345 (21%)	–			
			4 m	120	–	773 (20%)	64.6%			
			6 m	–	4.59	2115 (43%)	74.6%			
			9 m	–	5.42	2900 (60%)	–			
**13**	40+1	F	1 d	15	–	–	–	Transient idiopathic TCL	Yes, at 1 day old	Yes (6 months)/17 months
			11 d	12	–	–	–			
			17 d	–	4.29	1054 (69%)	65.8%			
			6 m	–	3.11	2539 (45%)	–			

GA*: Gestational Age; Age+ in d, days; m, months; y, year; TREC #: T-cell Receptor Excision Circle number, copies/μL, ALC^ Absolute lymphocyte count, x 10^6^/L, used as surrogate marker for lymphopenia; CD3+: CD3+ T cells absolute number and percentage; CD3+ or CD4+CD45RA+ naïve T cells as a percentage of total CD3+ or CD4+ T cells; TCL, T cell lymphopenia; ELBW, extremely low birth weight; SGA, small for gestational age; IUGR, intrauterine growth restriction; s/p, status post; GI, Gastrointestinal; BPD, bronchopulmonary dysplasia; IC, inconclusive or poor sample.

### Results and Outcomes of Positive Tests

Of the 9 term infants with abnormal TREC results (Patients 5-13), 5 had normal CD3+ lymphocyte counts for age on flow cytometry performed between 7 to 20 days of life (Patients 7-11), a median of 7 days after the initial TREC was done (range 4-10). 2 had mild T cell lymphopenia (TCL) (Patients 6 and 13) with CD3+ T-cells between 1000 to 1500 cells/µL, while 1 had severe TCL (Patient 12) with a CD3+ T cell count of 124 cells/µL (**Table 2**); all 3 had first flow cytometry performed between day 8-18 of life, a median of 6 days after the initial TREC was performed (range 6-16 days).

Interestingly, 4 infants (Patients 2-5) had a full blood count (FBC) on the same day as the initial TREC assay that showed low absolute lymphocyte counts consistent with an abnormal TREC result (**Table 2**). In infants where the TREC assay was repeated simultaneously with the eventual lymphocyte subsets done by flow cytometry, TREC count became normal when CD3+ T cell counts were normal in 3 patients (Patient 4, 8 and 11) and TREC counts were low where CD3+ counts T cell counts were low in 3 patients (Patient 3, 6 and 12). These findings indicated to us that the TREC counts were reflective of contemporaneous CD3+ T cell counts; hence, we considered the patients with abnormal TREC screening but eventually normal CD3+ lymphocyte counts on flow cytometry 7 to 20 days later, to have had transient idiopathic TCL that spontaneously resolved, rather than “false positive results” as referred to in earlier studies (11).

Of the 6 with normal lymphocyte counts on first flow cytometry, 2 were diagnosed with sepsis-related secondary TCL (Patients 5 and 11). Patient 5 had a low absolute lymphocyte count on the FBC done at birth which corresponded to the abnormal TREC result; repeat TREC was normal when sepsis had resolved. Patient 11 had been discharged at birth and sepsis was only diagnosed after the child was recalled for the abnormal TREC result and was found to have concomitant thrombocytopenia with a platelet count of 17 x 10^9^/L; both lymphopenia and thrombocytopenia resolved after antibiotic treatment. Patient 8 had a repeat TREC done simultaneously with the initial flow cytometry which improved from 6 copies/µL at day 3 of life to 75 copies/µL at day 13 of life, corresponding with the normal lymphocyte counts done at day 13. Thus, Patient 8 and 2 others (Patients 7 and 9) with normal lymphocytes on first flow cytometry were considered to have had transient idiopathic TCL rather than false positive results, while patient 10 had 22q11 Syndrome which may have contributed to initial transient TCL (**Table 2**). Of the 3 term newborns with abnormal lymphocyte counts on first flow cytometry, 2 had mild lymphopenia (Patients 6 and 13) with normal immunoglobulins and lymphocyte proliferation results (**Supplementary Table 1**). Patient 13’s lymphopenia spontaneously resolved by 6 months of age and was thus classified as transient idiopathic TCL, while Patient 6 still has persistent and worsening idiopathic TCL of unclear cause, with non-contributory PID gene panel and chromosomal microarray results (**Supplementary Table 1**).

One patient (patient 12) presented with an urgent positive TREC of 1 copy/µL and severe TCL with a CD3+ T cell count of 124 cells/µL, initially suspicious for SCID. Further workup revealed normal IgG, IgA and IgM, and normal lymphocyte proliferation to PHA, Concavalin A and anti-CD3 and anti-CD28. Genetic next-generation sequencing (NGS) panel for primary immunodeficiency and Whole Genome Sequencing did not reveal any significant pathogenic variants that could explain the severe lymphopenia (**Supplementary Table 1**). The infant remained well, had no complications from the BCG received at birth, and lymphopenia gradually improved and resolved by 6 months of age. Parents declined initial admission for isolation and prophylactic antibiotics as child was clinically very well with good growth and no infections, thus child was closely monitored outpatient instead. Rotavirus vaccination was omitted, while other routine non-live vaccinations were administered as usual. An unnecessary Hematopoietic Stem Cell Transplant (HSCT) was avoided, highlighting the importance of confirming the diagnosis of SCID with functional or genetic testing prior to HSCT.

All 4 extremely premature newborns (patients 1 to 4) had low absolute lymphocyte counts on FBC at birth, consistent with the low TREC counts done in the first week of life; TREC results improved with maturity. Lymphocyte counts eventually normalized in patients 1 and 4, while the other two died from complications of prematurity before the lymphocyte counts had fully normalized, but had near-normal CD3+ T cells of >1500 cells/µL prior to death from unrelated causes (**Table 2**).

The various diagnoses in the 13 patients were as follows (**Table 2**): 7/13 (54%) secondary TCL (2 patients with sepsis, 1 premature Trisomy 21 with hydrops and stoma losses, 1 premature Trisomy 21 with Chylothorax, 2 extremely premature, 1 partial 22q11 Syndrome) and 6/13 (46%) idiopathic TCL with no obvious cause. Of the 6 patients with idiopathic TCL, 5 (83%) were transient and resolved by 6 months, with 3 resolving within the first month, while 1 was persistent beyond 1 year. There were no patients diagnosed with SCID.

The overall incidence of clinically significant non-SCID TCL was 3 in 35888 or 1 in 11962 newborns. No cases of SCID were diagnosed clinically during this period in any of the hospitals in Singapore and for the 10 months following the data collection period up to time of writing in August 2021, indicating that the TREC screening was highly unlikely to have missed any cases of true SCID.

### BCG Vaccination Rates, Demographics, and Outcomes

Eleven of the thirteen patients with non-SCID TCL in our cohort received the BCG vaccination (Patients 1 and 4-13, **Table 2**), while 2 premature infants (Patients 2 and 3) died before vaccination could be given. Eight of 9 term infants (89%) received BCG vaccination within the first week after birth before the abnormal TREC result returned (Patients 6-13) with all receiving BCG vaccination within the first 3 days of life except patient 10 who was vaccinated at 6 days old. One term infant (Patient 5) with sepsis and lymphopenia diagnosed on the first day of life received BCG on day 21 prior to discharge after completing intravenous antibiotics and normalization of TREC results (parents declined initial lymphocyte subset testing). The 2 premature infants who survived to discharge (Patients 1 and 4) were given BCG vaccination prior to discharge after the lymphopenia had resolved.

The 8 term infants were given BCG vaccine at a median of 2.5 days old (range 1-6 days) while their TREC counts were low (median 9.5 copies/uL, range 6-15 copies/uL). 5 had normal T lymphocyte counts of more than 1500 CD3+ T cells/uL on first flow cytometry (patients 7-11) performed between day 7 to 19, while 3 had TCL on first flow cytometry (Patients 6, 12 and 13) performed between day 8 to 18.

Patient 6 had initial CD3+ 1043 cells/uL at day 18 of life and persistent idiopathic TCL of unknown cause even at 10 months old despite genetic testing; Patient 12 had initial severe TCL with CD3+ 124 cells/uL at 8 days of life and CD3+ 773 cells/uL at 4 months old, which eventually resolved with CD3+ 2115 cells/uL at 6 months old but no cause was found for the transient idiopathic TCL despite genetic testing; Patient 13 had CD3+ 1054 cells/uL at 17 days old which resolved by 6 months of age and genetic testing was not performed for this transient idiopathic TCL.

Although 5 patients had normal T cell counts on first flow cytometry, one of these (Patient 11) was diagnosed with sepsis-related secondary TCL associated with thrombocytopenia, and at the time that BCG was given when TREC count was low, there was likely to have been true lymphopenia although a full blood count was not done at birth thus no confirmation is possible. Patient 11 and Patient 8 had repeat TREC counts done at the same time as first flow cytometry, with normalization of TREC counts when CD3+ counts were normal, indicating that the initial low TREC count at the time of BCG vaccination likely reflected an initial true albeit transient TCL, rather than a false positive result.

There were no short to medium term BCG-related complications in any of the newborns on follow-up for at least 10 months after the completion of recruitment (median 17 months, range 10 to 22 months, excluding deceased extremely premature patients). This was despite low TREC counts (median 9.5, range 1 to 15) at time of BCG vaccination in 8 patients, (**Table 2**), documented low initial CD3+ T cell counts of between 124 cells/µL to 1054 cells/µL soon after BCG vaccination in 3 patients (Patients 6, 12 and 13), and persistent significant lymphopenia in patient 12 (CD3+ T cell count 773 cells/µL at 3 months) and patient 6 (CD3+ T cell count 683 cells/µL at 10 months).Longer follow-up is needed to determine if the transient TCL during the neonatal period has any long-term relation to the development of late BCG complications such as osteomyelitis.

## Discussion

We have reported the Singaporean experience of implementing universal Newborn Screening for Severe Combined Immunodeficiency (SCID) using detection of T-cell receptor excision circles (TREC) *via* the commercial Enlite Neonatal TREC Assay, while retaining the practice of concomitant routine BCG vaccination at birth in view of tuberculosis (TB) endemicity. Using a threshold for normal TREC to be >18 copies/µL, we had an overall retest rate of 0.1%, repeat sampling rate of 0.06%, and a referral rate to immunologist of 0.04%. In 1 year, we detected 13 newborns with non-SCID T-cell lymphopenia (TCL), but no SCID cases. Only 3 cases had clinically significant TCL beyond 1 month, with an overall incidence of 1 in 11962 newborns. All 9 term infants with abnormal TREC results received BCG vaccination in the neonatal period, with 89% (8/9) receiving it within the first 6 days of life while TREC and lymphocyte counts were low; none have had complications on median follow-up of 17 months. To our knowledge, we are the first country to implement a national newborn SCID screening algorithm in a setting of high TB endemicity and routine neonatal BCG vaccination.

The results we obtained with the Enlite Neonatal TREC kit were comparable to those of previously published reports using the same kit, with reported recall rates from 0.005% - 0.09% for large cohorts (**Table 1**) [France: (12); Catalonia: (11); Taiwan: (15); California: (10); Israel: (13)]. Our overall incidence of 1 in 11962 newborns is consistent with other published results where incidence ranges from 1 in 337 in France to 1 in 19952 in California, depending on the cutoff used to define mild lymphopenia (10, 12, 29). The use of a commercially available Enlite Newborn TREC assay obviated the need to locally develop and validate an assay *de novo* for a rare disease in a small population, and enabled rapid national implementation with standardization and reproducibility according to ISO15189 standards. In addition, the single-step DNA elution and gene amplification process bypasses the DNA extraction step, thus the consent-taking process could be simplified and genetic counselling omitted. Similar technology could potentially be used for screening of other genetic conditions such as Spinal Muscular Atrophy and X-linked Agammaglobulinemia in future (30).

In the first year of screening in Singapore, 13 of 35888 newborns screened positive and required flow cytometric evaluation. All 13 newborns had non-SCID TCL, and only 1 newborn had significant, severe TCL with CD3+ T cells <300 cells/µL, which eventually spontaneously resolved by 6 months of age. Having a category of urgent positive at TREC <4 accurately identified the one patient with severe TCL (CD3+ T cells 124 cells/µL) and a higher risk of true SCID, differentiating that patient from the other 12 with milder non-SCID TCL and facilitating earlier evaluation. Non-SCID TCL makes up more than 90% of all cases of abnormal TCL detected by TREC screening and can be secondary to sepsis, third-space losses, prematurity, syndromes, and idiopathic TCL (10, 26). On detection of an abnormal TREC, the priority should be to determine the severity of the TCL and likelihood of SCID with flow cytometry lymphocyte subsets and naïve and memory T cell proportions, to risk-stratify and guide further investigations and management. Since the majority of infants with abnormal TREC results have normal immunoglobulin levels and lymphocyte proliferation to PHA (11), and due to concerns of large blood volumes required and cost of testing, additional immunological evaluation was only performed for those with either 1) severely low TCL CD3+ <300 cells/µL 2) persistently abnormal TREC results beyond 1 month of life or 3) those who presented with other clinical concerns. This approach is more cost-effective and resource-efficient in countries where there is limited access to functional immunological testing.

Inoculation of live BCG at birth in TB endemic countries like Singapore poses a risk of disseminated BCG disease in the small number of SCID patients who are yet to be diagnosed (17, 31, 32) Careful family history-taking prior to neonatal vaccination with BCG may detect the 20% of SCID cases with a positive family history (4), but most SCID patients would still be missed. Delaying the BCG vaccine beyond the first month of age may diminish the BCG-associated morbidity and mortality in SCID patients (20).

However, delayed BCG vaccination may have unintended consequences on the majority of the population. It may result in a decline in BCG coverage from the current 99% (18) due to the “missed opportunity” of vaccinating patients at birth (33), leading to an increase in TB meningitis and military TB cases. BCG vaccine-related adverse events may also increase as intradermal inoculation of BCG is difficult and may be administered incorrectly (34) by less experienced staff who have to administer delayed BCG vaccination outside of hospital nurseries. In Taiwan, delaying the BCG vaccination from birth to between 1-4 weeks resulted in a decrease in the rate of BCG vaccination completion by the age of 1 month from 80% before 2010 to 53% in 2014 (15). However, there was also a decreased incidence of tuberculosis from the previously reported 70 cases per 100,000 population in 2009 to 48 cases per 100,000 population in 2014 (15, 35, 36), thus the incidence of TB meningitis remained between 1-2 cases annually. In places with moderate to high tuberculosis burden, the risk and benefit of the “screen-and-delay” strategy is uncertain, especially as SCID is a comparatively rare disease with an incidence of between 1 in 22,000 to 130,000 based on newborn screening (29). The incidence of SCID in Singapore is around 1 in 100,000 newborns based on historical clinical data (37) and recent unpublished data.

In the rare BCG-vaccinated SCID patients, BCG complications typically develop at a median age of 4 months (31, 32). In our recently published 15-year experience with disseminated BCGosis (19), we had 4 patients with SCID who first presented with BCG-related complications between 1.5 to 5.5 months of life [**Supplemental Table 2**]. Since we have been able to complete initial lymphocyte evaluation for all patients with abnormal TREC results by 21 days of life, and full immunological investigations by 6 weeks of life, we would have a window of intervention between the diagnosis of SCID and development of BCG complications to start antibiotic prophylaxis and prepare patients for curative HSCT. This same pre-emptive treatment strategy could be applied to patients with non-SCID TCL picked up on TREC screening, who may also be at higher risk for disseminated BCG disease.

No significant complications arose from BCG vaccination in our cohort who were given BCG vaccination while TREC was low, for at least 10 months after the completion of recruitment despite persistent lymphopenia in 3 patients on follow-up. Longer follow-up is needed to determine if the transient T lymphopenia during the neonatal period has any long-term relation to the development of late BCG complications such as osteomyelitis. It is important to note that Adenosine Deaminase (ADA) deficiency and leaky variants of SCID may present later in life and require eventual HSCT, but may have normal lymphocyte subsets and proliferation at initial screening (38, 39). Thus, as an added precaution for patients with non-SCID TCL who have received BCG vaccination at birth, longer term follow up is needed to monitor for later manifestations of SCID and disseminated BCG disease.

The main limitation in our study is the relatively small number of newborns screened due to the small population of Singapore of 5.7 million, and short follow-up time of 1 year after the end of the recruitment period. However, our non-SCID TCL detection rate and recall and referral rates are similar to that of other larger studies in France (12); Catalonia (11); Taiwan (15); California (10); and Israel (13) (**Table 1**) which supports the validity of our methods. We plan to follow this study up with a larger number of newborns screened over a longer period of time in Singapore. In terms of the strategy of retaining BCG vaccination at birth, we did not have any diagnosed SCID patients who received the BCG vaccine to directly validate the safety of our approach. However, our experience of previous SCID patients who received BCG vaccination at birth (**Supplemental Table 2**) gives us confidence that early diagnosis of SCID and early prophylactic antibiotics for BCG is a reasonable approach, given the rarity of SCID and endemicity of TB in Singapore.

## Conclusion

To conclude, Singapore has successfully implemented newborn screening for SCID in 2019 using the commercially available Perkin Elmer Enlite Neonatal TREC Assay kit to quantify TRECs in dried blood spots as part of the Expanded Newborn Screening Program, while retaining the practice of concomitant routine BCG vaccination at birth. A cut-off threshold of <18 copies/µL yielded acceptable retesting and recall rates similar to previously published data, and was effective in detecting T cell lymphopenia (TCL) with no missed cases of SCID thus far.

A large proportion of positive cases have transient idiopathic TCL which spontaneously resolves with no significant clinical sequelae, thus an FBC with differential and lymphocyte subsets is sufficient for initial testing, with more detailed testing of immunoglobulins and lymphocyte proliferation reserved for patients with severe or persistent TCL or other clinical concerns.

Newborn screening for SCID can be implemented despite almost universal BCG vaccination, and our results provide evidence that patients with transient TCL and no significant underlying primary immunodeficiency are able to tolerate BCG vaccination with no short/medium-term complications.

Longer duration of TREC screening is needed to define the exact incidence of SCID in Singapore, and to determine if the transient TCL observed during the neonatal period has any long-term significance.

## Data Availability Statement

The raw data supporting the conclusions of this article will be made available by the authors upon request.

## Ethics Statement

Ethical review and approval was not required for the study on human participants in accordance with the local legislation and institutional requirements. Written informed consent from the participants’ legal guardian/next of kin was not required to participate in this study in accordance with the national legislation and the institutional requirements.

## Author Contributions

SC and YZ designed the study and organised the database. SC, WL, YZ, CC, KCT, MS, and ET developed protocols for management of abnormal TREC results and plans for antibiotic prophylaxis of BCG disease. SP and SL implemented, validated and ran the TREC assays. SC, YZ, KLT, JS, and WL collected the clinical data, while SP and SL collected the laboratory data. Results were analysed and interpreted by SC, YZ, WL, and TA. SC wrote the first draft of the manuscript. SL and YZ wrote sections of the manuscript. All authors contributed to manuscript revision and read and approved the submitted version.

## Funding

KK Women’s and Children’s Hospital, Division of Pediatric Medicine Subspecialties Education, Training and Research Funds were used for open access publication fees.

## Conflict of Interest

The authors declare that the research was conducted in the absence of any commercial or financial relationships that could be construed as a potential conflict of interest.

## Publisher’s Note

All claims expressed in this article are solely those of the authors and do not necessarily represent those of their affiliated organizations, or those of the publisher, the editors and the reviewers. Any product that may be evaluated in this article, or claim that may be made by its manufacturer, is not guaranteed or endorsed by the publisher.

## Abbreviations

BCG, Bacille-Calmette-Guerin; CDC, Centers for Disease Control and Prevention; HSCT, Hematopoietic Stem Cell Transplant; NBS, Newborn Screening; NGS, Next Generation Sequencing; PID, Primary Immunodeficiency Disease; SCID, Severe Combined Immunodeficiency; SD, Standard Deviation; TB, Tuberculosis; TCL, T-cell Lymphopenia; TREC, T-cell Receptor Excision Circle; USA, United States of America.
