# Supplementary material for: Implementation of Universal Newborn Screening for Severe Combined Immunodeficiency in Singapore While Continuing Routine Bacille-Calmette-Guerin Vaccination Given at Birth
Source: Front Immunol. 2022 Jan 3;12:794221. doi: 10.3389/fimmu.2021.794221 (PMC8761728; doi:10.3389/fimmu.2021.794221)
Supplement: Supplementary file 1 [file Table_1.docx]

Supplementary Material

## Supplementary Methods

**TREC Assay Validation**

Calibration curves were constructed by the Enlite Workstation software using blanks and three levels of TREC/ beta-actin DBS calibrators (C1,C2,C3) performed in assay triplicates on each plate. Fluorescence excitation at central wavelength of 320nm and emission counts and intensity measured at 615nm, 665nm, and 780nm was directly related to the number of TREC or beta-actin copies/µL of blood. For the corrected results, the TREC and beta-actin levels of the samples were fitted against the ArcSinh transformed concentrations of the calibration spots by using un-weighted linear regression. The correlation coefficients of the calibration curve of 16 runs performed were typically above 0.996 for TREC and 0.995 for beta-actin. The precision of the TREC assay as determined from 30 runs using the control samples (C1, C3) was within the acceptable limit of the reagent lot variation (i.e. C1 [SD (Ln)=0.49] and C3 [SD (Ln)=0.55] was within the calculated tolerance limit of 0.86 and 0.94 respectively). Intra-assay variation results obtained were consistent with manufacturer’s targets with the C1 control of 0.55 Ln copies/µL and C3 control of 0.63 Ln copies/µL. Average based inter-assay variation of the C1 and C3 control was 0.49 Ln copies/µL and 0.55 Ln copies/µL respectively. The TREC median of the C2 control was 0 copy/µL while 96.7% of all measured values presented ≤5 copies/µL.

## Supplementary Tables

**Supplemental Table 1. Result of Additional Immunological Evaluations**

| **Case** | **GA*** | **Age at subsets** | **CD3+ cells/**µL **(%), at age** | **Immunology Test Results** |
| --- | --- | --- | --- | --- |
| **1** | 26+2 | 78 d  16 m | 633 (36%)  2157 (60%) | IgG normal |
| **3** | 29+1 | 28 d  43 d  57 d | 1265 (73%)  893 (66%)  2096 (53%) | IgG, IgA, IgM, IgE normal initially  Later IgG undetectable just before demise |
| **4** | 32+2 | 29 d | 2149 (65%) | IgG, IgA, IgM, IgE normal |
| **6** | 37+6 | 18 d  5.5 m  10 m | 1043 (53%)  881 (39%)  683 (44%) | IgG, IgA, IgM, IgE normal  Lymphocyte Proliferation^ normal  Genetic PID panel and CMA no obvious cause |
| **10** | 39+1 | 19 d | 2248 (51%) | IgG high, IgA, IgM, IgE normal  Antenatally confirmed 22q11 deletion |
| **11** | 40+0 | 7 d | 3093 (67%) | IgG, IgA normal, IgM high  blood and urine CMV PCR test negative |
| **12** | 40+0 | 8 d  14 d  62 d  3 m  5.5 m  9.5 m | 124 (12%)  210 (15%)  345 (21%)  773 (20%)  2115 (43%)  2900 (60%) | Initially high IgM and IgE, normal IgG, IgA  Later persistently high IgGAME  Lymphocyte Proliferation^ normal  Genetic PID panel, Whole Genome Sequencing no obvious cause |
| **13** | 40+1 | 17 d  6 m | 1054 (69%)  2539 (45%) | IgG normal  Lymphocyte Proliferation^ normal |
| GA*: Gestational Age; CD3+: CD3+ T cells absolute number and percentage; Lymphocyte proliferation^ to Phytohemagglutinin, Concavalin A and anti-CD3 anti-CD28 stimulation, PID primary immunodeficiency commercial gene panel. IgGAME Immunoglobulins G, A, M, and E; CMV cytomegalovirus, PCR polymerase chain reaction test. d day, m months | | | | |

**Supplemental Table 2. Age at presentation of BCG symptoms for previous SCID patients in Singapore with Disseminated BCG Disease (modified from Ong R et al 2020)(19)**

| **Case** | **Sex** | **PID Diagnosis and Clinical Phenotype** | **Age of PID Diagnosis (months)** | **Presenting symptoms** | **Age of first BCG symptoms (months)** | **Extent of BCG complications** | **HSCT** | **Outcome** |
| --- | --- | --- | --- | --- | --- | --- | --- | --- |
| **1** | Male | X-SCID (T-, B-, NK-) with B cell lymphoproliferative disease  IL2RG mutation | 5.5 | Left humerus BCG osteomyelitis, parainfluenza 1 and *Aspergillus fumigatus* bronchopneumonia | 5.5 | Left humerus osteomyelitis at 5.5 months with lung, spleen and liver granulomas. Right foot BCG abscess at 1 year old | Yes | Survived |
| **2** | Female | SCID (T-, B+, NK-), but negative for JAK3 and IL7R mutations | 2.1 | Left hip MSSA and BCG septic arthritis, MSSA bacteremia, Right lung abscess, BCG bacteremia with infective endocarditis | 1.5 | BCG pulmonary valve infective endocarditis, BCG bacteremia and BCG septic arthritis at 1.5 months | Yes | Survived |
| **3** | Female | SCID (T-, B+, NK+), IL7R mutation | 5.5 | Left inguinal BCG lymphadenitis and BCG site ulceration with fever at 4 months old | 4.5 | Left inguinal lymphadenitis, BCG site ulceration, Left pelvic and psoas abscess, spleen microabscesses | Yes | Died from Graft vs Host disease |
| **4** | Male | SCID (T-, B-, NK-), agammaglobulinemia, impaired lymphoproliferation, genetics negative | 7.3 | BCG injection site ulceration and abscess at 4 months old, prolonged fever at 6 months old | 4 | Left gluteal BCG wound abscess, splenic abscesses at 7 months old, thigh and skin BCG abscesses with liver and bone lesions and pulmonary BCG infection at 1.3 years. | No | Died from BCG disease |
| PID Primary immunodeficiency Disorder. BCG Bacille-Calmette-Guerin vaccine HSCT Hematopoietic stem cell transplant. MSSA Methicillin Sensitive *Staphylococcus Aureus*. BCGosis Disseminated BCG infection. T: T cells, B: B cells, NK Natural Killer cells, - absent, + present. JAK3 Janus Kinase-3, IL7R interleukin 7 Receptor, X-SCID X-linked SCID, IL2RG Interleukin 2 Receptor subunit gamma | | | | | | | | |
